# Clinical course of non-small cell lung cancer patients with dry pleural dissemination: Retrospective observational study

**DOI:** 10.1097/MD.0000000000019533

**Published:** 2020-03-13

**Authors:** Woojung Kim, In Kyu Park, Samina Park, Chang Hyun Kang, Young Tae Kim

**Affiliations:** a Department of Thoracic and Cardiovascular Surgery, Seoul National University Hospital; b Department of Thoracic and Cardiovascular Surgery, Seoul National University College of Medicine; c Seoul National University Cancer Research Institute, Seoul, Republic of Korea.

**Keywords:** malignant pleural effusion, neoplasm seeding, non-small cell lung cancer

## Abstract

We investigated the prognosis of patients with dry pleural dissemination (DPD) of non-small cell lung cancer (NSCLC) and the risk factors of developing to malignant pleural effusion (MPE).

We retrospectively reviewed 104 patients with NSCLC and DPD, confirmed surgically from 1996 to 2016. Incidence rate and risk factors of MPE were analyzed statistically. The prognosis of NSCLC patients with MPE was evaluated using the Kaplan–Meier method.

The most common histologic type was adenocarcinoma in 95 (91.3%) patients. The median follow-up duration was 65.5 months and the median survival time was 37.7 months. MPE developed in 51 (49%) patients, and the median effusion-free interval was 41.9 months. The median survival time of the patients with and without MPE was not different (41.3 vs 31.7 months, *P* = .16). No predictive factors for the development of MPE were identified. Fifteen (14.4%) patients underwent invasive procedures for the management of MPE.

Almost half of all patients with NSCLC and DPD experienced MPE, and 14.4% patients developed symptomatic MPE requiring invasive procedures. MPE in DPD did not affect the survival in NSCLC patients.

## 1 Introduction

Pleural dissemination is one of the important modes of metastasis and a poor prognostic factor of non-small cell lung cancer (NSCLC).^[1,2]^ Although every pleural dissemination is classified as the same stage, M1a, pleural dissemination is not a single entity of disease but a spectrum of disease with various tumor burden encompassing from limited pleural seeding to overt malignant pleural effusion (MPE).^[3,4]^ Dry pleural dissemination (DPD) is a relatively early stage of pleural dissemination defined as pleural seeding without effusion and can be detected radiologically or surgically in a patient with clinically resectable lung cancer. DPD is detected in 1.2% to 2% of clinically resectable lung cancer patients.^[5,6]^ A few studies showed relatively better prognosis of DPD compared with MPE and several studies reported favorable outcome after pulmonary resection in patients with surgically diagnosed DPD.^[4–6]^ However, the prognosis of DPD regarding the development of MPE and survival, especially in the era of targeted therapy, is still unclear.

Another clinical issue is how to manage patients with DPD. DPD does not induce any symptoms and specific treatment directed to DPD itself is not necessary.^[7]^ However, it can progress to MPE which impairs quality of life and requires invasive procedures. If DPD is detected intraoperatively in lung cancer patients, there is a chance to perform any intervention to prevent MPE. Intraoperative talc poudrage is a suggestive modality to prevent MPE, and the thoracoscopic talc poudrage has been reported to be the most effective method to prevent recurrence of MPE in a patient with expandable lung.^[8–11]^ We investigated the pattern of progression of DPD to MPE and the prognosis of NSCLC patients with surgically confirmed DPD.

## 2 Materials and methods

A total of 104 NSCLC patients with surgically confirmed DPD from February 1995 to March 2016 were reviewed retrospectively. All patients had a pathological diagnosis of pleural seeding of primary NSCLC. Patients with MPE diagnosed preoperatively or related with combined malignancies other than NSCLC were excluded. The data about age, sex, body mass index, histologic type, clinical stage based on the 8th edition of the TNM classification, location of tumor, visceral pleura invasion (operation finding), underlying diseases, presence of epidermal growth factor receptor gene (EGFR) mutation or echinoderm microtubule-associated protein-like 4 (EML4) – anaplastic lymphoma kinase (ALK) gene fusion, extent of tumor resection, oncologic treatment, recurrence, the presence of MPE, and survival were obtained by reviewing medical records. The effusion-free interval (EFI) was defined as the duration from the day of operation to the day of radiologic detection of MPE. The overall survival was defined as the duration from the day of operation to the day of death by any causes or the day of the last follow-up in months. Effusion-free survival was defined as the duration of survival without evidence of MPE. Resection of the primary tumor in patients with limited DPD was decided by the surgeon considering the location of the tumor and the pulmonary function. Talc poudrage was not performed in the study cohort. First-line oncologic treatments consisted of platinum-based cytotoxic chemotherapy and tyrosine kinase inhibitor (TKI). Surveillance after treatment was conducted by chest x-ray and computed tomography. Positron emission tomography was performed when recurrence was suspected clinically or radiologically. The last follow-up date was August 30, 2018.

### 2.1 Malignant pleural effusion

MPE was diagnosed radiologically or histologically. In patients with a large amount of effusion or respiratory symptoms related with effusion, therapeutic interventions including thoracentesis or invasive intercostal drainage (percutaneous catheter drainage or tube thoracostomy) were performed, and the MPE was confirmed histologically. In the patients with a small amount of effusion without symptom, MPE was diagnosed radiologically, and the intervention and subsequent cytologic diagnosis were exempted.^[7]^ The pleural effusion developed in the immediate postoperative period was not counted as MPE.

### 2.2 Statistical analyses

**Figure 1:**
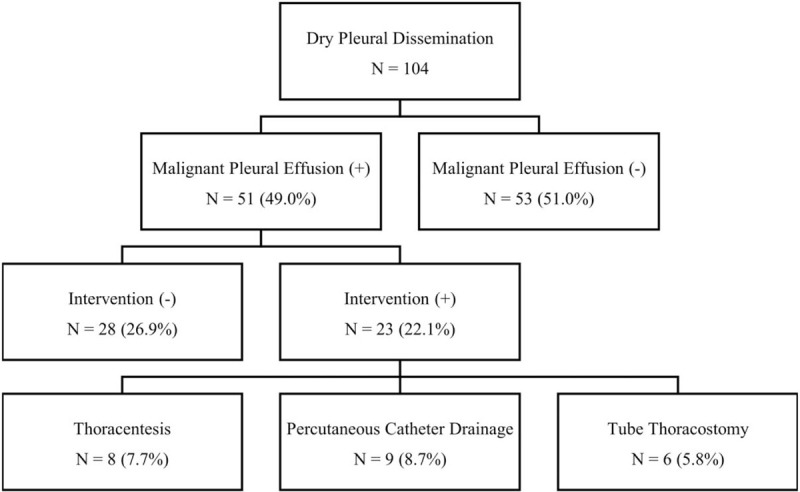
Flow diagram for the development and management of malignant pleural effusion.

Follow-up duration was calculated using the reverse Kaplan–Meier method. The effusion-free survival time and overall survival time were calculated using the Kaplan–Meier method. Prognostic factors for EFI were evaluated using the log-rank test for univariable analysis. Parameters showing *P* ≤ .2 in univariable analyses were included in the multivariable analysis conducted by the Cox proportional hazard model. Prognostic effects of age (≤60 vs >60), sex, smoking status (never vs ever), histologic type (adenocarcinoma vs others), clinical T stage (T1/2 vs T3/4), tumor resection, visceral pleural invasion, targetable mutations, and TKI therapy on EFI were tested. Patients were divided into 2 groups (Fig. 1); MPE group vs no-MPE group, and the survival and the baseline patient's characteristics of 2 groups were compared. The Student *t* test was used for the comparison of continuous variables, and Chi-Squared method was used for the comparison of discrete variables. The variables with *P* value less than .05 were considered statistically significant. Post-MPE survival time was from the date when the patient was diagnosed with MPE to death or last follow-up date. Statistical analyses were performed using the Statistical Package for Social Science (ver. 21.0, IBM Corp., Armonk, NY). This study was approved by the Institutional Review Board of Seoul National University Hospital, Seoul, Korea (approval number: H-1805-141-948). This study complied with the Declaration of Helsinki.

## 3 Results

The mean age was 60.9 ± 12.5 years (range: 30–86 years), and 56 patients (53.8%) had pleural nodules in computed tomography. Adenocarcinoma (n = 95, 91.3%) was the dominant histology and 42 (40.4%) patients had targetable mutations (EGFR mutation – 37, EML4-ALK gene fusion – 5). Platinum-based cytotoxic chemotherapy was the standard first-line regimen and was performed in 77 patients (74.0%). TKIs were used as a first-line regimen in 27 patients (26.0%) with targetable mutation and as a second-line regimen in 13 patients (12.5%) with the targetable mutation. Additional 12 patients (11.5%) without the targetable mutation had TKI as a salvage treatment for a brief period. Two patients with EML4-ALK gene fusion did not have TKI therapy. Baseline characteristics of the patients are described in Table 1.

**Table 1:**
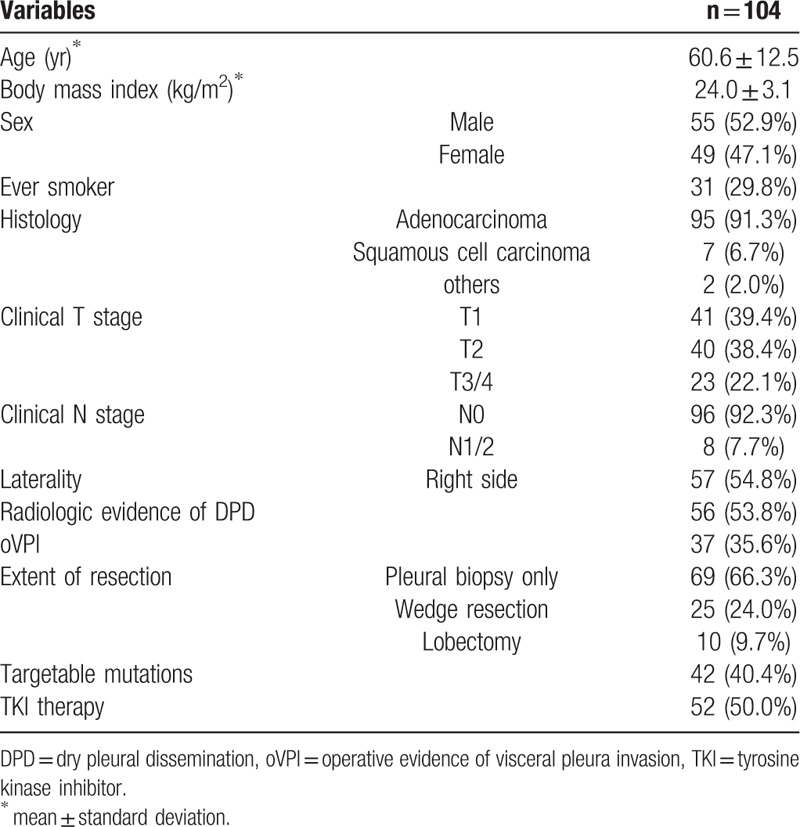
Patients’ characteristics.

The median follow-up duration was 65.5 months and the median overall survival time was 37.7 (interquartile range [IQR]: 22.2–64.9) months. The overall survival rates at 3-year and 5-year were 51.4% and 29.3%, respectively.

MPE developed in 51 (49.0%) patients (Fig. 1). The median EFI was 41.9 (IQR: 20.5–61.6) months. The median effusion-free survival time was 26.7 (IQR: 13.0–41.9) months, and the 5-year effusion-free survival rate was 9.0%.

**Figure 2:**
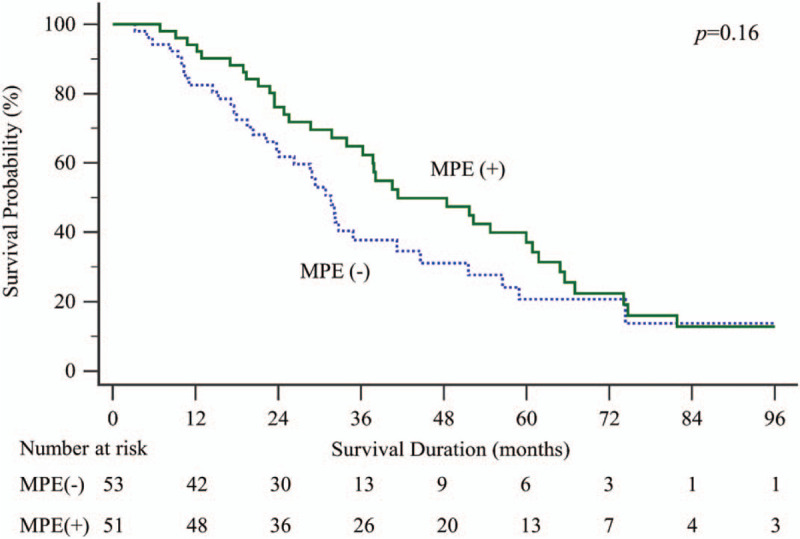
The Kaplan–Meier curves for overall survival according to the development of malignant pleural effusions.

None of the preoperative and operative factors were predictive for the EFI except tumor resection in univariable analyses (Table 2). In multivariable analysis, however, tumor resection was also not a significant predictor for the EFI (Table 3). The overall survival of the MPE group and no-MPE group was not different statistically (MPE: median = 41.3 [IQR: 25.8–67.0] months, no-MPE: median = 31.7 [IQR: 17.6–56.5] months, *P* = .16) (Fig. 2). Table 4 shows the comparison of preoperative and operative factors between the MPE group and no-MPE group.

**Table 2:**
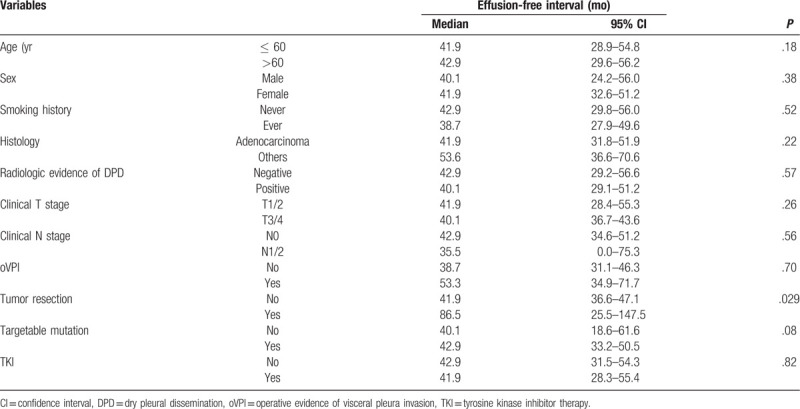
Univariable analysis for the risk factors of an effusion-free interval.

**Table 3:**
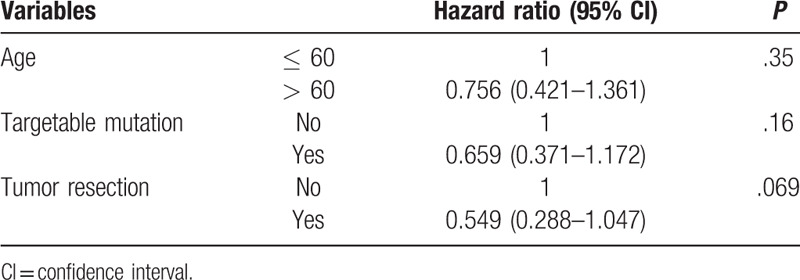
Multivariable analysis for risk factors related to an effusion-free interval.

**Table 4:**
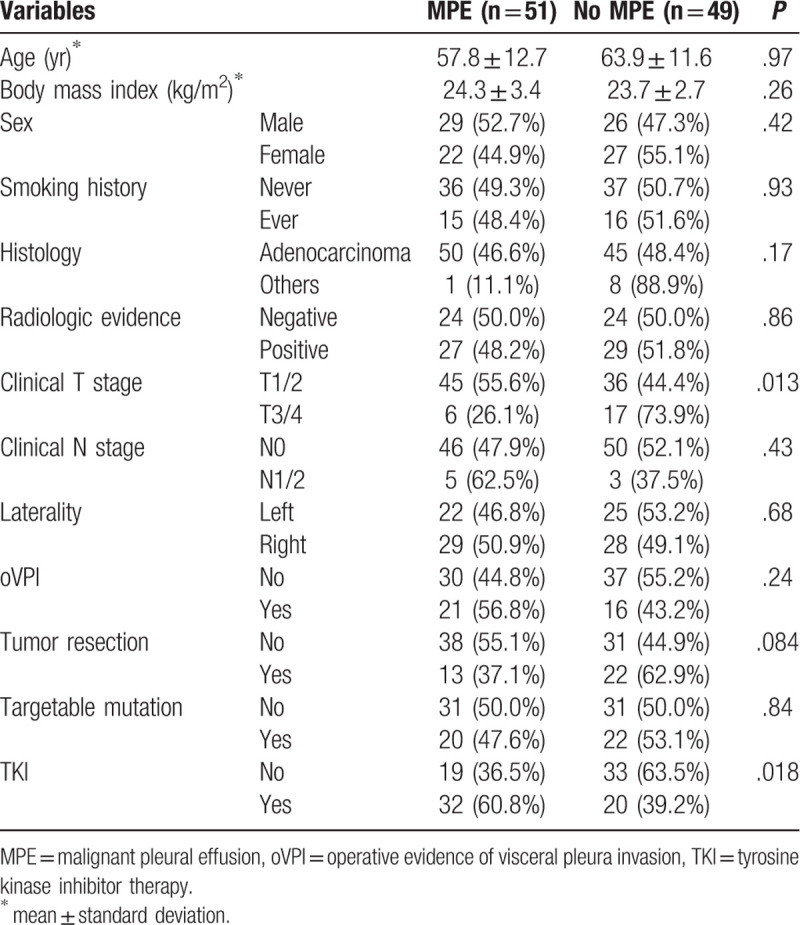
Comparison of patients with or without malignant pleural effusions.

Among 51 patients with MPE, 23 (22.1%) patients underwent interventions for the management of MPE and subsequently got the cytologic diagnosis of MPE. Eight (7.7%) patients were treated with a single episode of thoracentesis. Invasive intercostal drainages were performed in 15 (14.4%) patients, percutaneous catheter drainage in 9 (8.7%) patients, and tube thoracostomy in 6 (5.8%) patients (Fig. 1). The median survival time after diagnosis of MPE (post-MPE survival) in all MPE patients was 8 (IQR: 3.8–21.8) months and the post-MPE survivals of patients with the intervention (cytologic confirmation) and without intervention (no cytologic confirmation) were not different statistically (intervention group: median = 12.5 [IQR 4.9–26.8] months, no-intervention group: median = 5.8 [IQR 2.6–16.0] months, *P* = .87).

## 4 Discussion

The present study showed that 49.0% of NSCLC patients with surgically confirmed DPD eventually experienced MPE during their respective disease courses. Further, 14.4% of patients needed invasive procedures for the management of MPE.

DPD is an earlier manifestation of pleural dissemination of NSCLC and can be detected radiologically, or surgically in patients without access to radiologic imaging. The reported prognosis of patients with intraoperatively diagnosed DPD is relatively favorable. Okamoto et al reported that the median survival time was 25.9 months and the 5-year survival rate was 23.7% in patients with intraoperatively diagnosed DPD.^[12]^ The present study showed a similar result. The median survival time was 37.7 months, and the 5-year survival rate was 26.3%. A recent study by Li et al, which enrolled patients with lung adenocarcinoma, treated since 2006, showed better results. In Li's study, the 3-year overall survival rate was 69.2%.^[4]^ The improved survival in recently treated patients might be related to the advancement of TKIs that target the EGFR gene mutation.^[13,14]^ Approximately 60% of patients in Li's study had an EGFR mutation. In our study, only 40% of patients had targetable mutations (EGFR mutation or EML4-ALK gene fusion) and the median survival time of patients with the targetable mutation was longer than that of patients with wild-type genes (52.3 vs 34.8 months).

MPE is an advanced form of pleural dissemination in NSCLC.^[1]^ MPE reduces the quality of life and requires invasive procedures.^[7,15,16]^ Its prevalence is approximately 16% at initial diagnosis in patients with advanced NSCLC. Finally, 40% of patients with NSCLC experience MPE during their lifetimes and approximately half are treated invasively.^[17,18]^

There are few reports about the relationship between DPD and MPE. Kim et al reported that MPE developed in all 19 patients with radiologic DPD, and the mean interval to MPE was 19 ± 16.7 months.^[19]^ Li et al reported that MPE developed in 18.6% of 43 patients with intraoperatively diagnosed DPD (without radiologic evidence) during the 3-year follow-up period.^[4]^ In the present study, MPE developed in 49% of patients with DPD of NSCLC and the median interval to MPE was 42 months. The moderate prevalence rate of MPE in the present study might be due to our decision to include patients with DPD with and without radiologic evidence.

We investigated predictive factors for MPE in patients with DPD to guide preventive treatment for MPE. No clinicopathologic factors predicted the development of MPE. Wu et al studied the association of lung adenocarcinoma and MPE and reported that patients with MPE at initial diagnosis had more EGFR gene mutations than a patient who had MPE after disease progression.^[16]^ In our study, adenocarcinoma was not associated with EFIs. Also, the EGFR gene mutation and TKI therapy were not associated with EFIs. Tumor burdens of patients with radiologically positive DPD and radiologically negative DPD were different. However, the presence of radiologic evidence of DPD was not associated with EFIs (*P* = .61). Resection of the tumor was performed in 33.7% of patients and was a significant predictive factor for EFIs following univariable analysis (which lost power during multivariable analysis). A reasonable explanation for this finding could be that tumor resection may have prevented or delayed further intracavitary progression.^[5]^ However, it is premature to recommend preventive tumor resection because it was not a significant factor and tumor resection was decided subjectively considering tumor size and location, pulmonary function, node metastasis status, and extent of pleural dissemination.

The MPE and no-MPE groups showed statistically similar overall survival with a trend of more prolonged survival in the MPE group (41.3 vs 31.7 months, *P* = .16). This finding suggests that the patient who succumbs to death by systemic progression of a disease other than MPE would be less likely to experience MPE. Between-group comparisons presented in Table 4 show that patients with relatively poor prognosis experienced less MPE. Patients with advanced T stage and without TKI therapy had less MPE than patients with early T stage and TKI therapy.

MPE induces respiratory symptoms, decreases quality of life, and is associated with significant social and medical costs.^[20]^ It would be beneficial if a simple procedure could prevent MPE because many patients with DPD are at risk for developing MPE. The talc poudrage is the most effective method for managing symptomatic MPE and can be performed simultaneously as the patients are confirmed with DPD intraoperatively. The reported success rate of talc poudrage in patients with MPE related to lung cancer is between 82% and 91%.^[8–11]^ Although disease status and the condition of patients with symptomatic MPE and asymptomatic DPD are different, the efficacy of talc poudrage in DPD may be similar to that of symptomatic MPE. Therefore, prophylactic talc poudrage may be considered to prevent the development of MPE in patients with DPD of NSCLC. In this study, however, the actual proportion of patients who needed an invasive procedure for MPE was 14.4% in their course of disease. In other words, the potential beneficiary of preventive intraoperative talc poudrage was 14.4% in patients with DPD. Thus, we think intraoperative prophylactic talc poudrage in NSCLC patients with DPD is not recommended. Furthermore, there was no predictive factor for the development of MPE.

This study had several limitations. First, this was a single institutional retrospective study and the number of cases was not enough to provide a higher level of evidence. However, it should be noted that all the patients suffering from DPD were reviewed, and this study can provide meaningful understanding about the fate of DPD in the real world. In order to obtain higher level of evidence, a multi-institutional prospective cohort study should be conducted. Secondly, modalities for the management of MPE had been selected largely based on the patient's subjective symptoms and this strategy might have resulted in selection bias. However, symptom-based management is a generally recommended strategy^[7]^ and it is an intrinsic limitation of studies about management of MPE. Thirdly, the study duration was long, and oncologic treatments evolved rapidly during the study period. Thus, we suspect that patient outcomes would be much better in recent patients. Finally, we considered any asymptomatic effusions as MPEs, although cytologic examination was omitted. Effusions in patients with NSCLC can be paramalignant, and our assumption is subject to criticism.^[21,22]^ However, all patients with asymptomatic effusions in this study initially had pathologically proven DPD. Their post-effusion survival was similar to patients with cytology-positive MPE. Therefore, the definition of MPE in this study may be appropriate.

## 5 Conclusion

MPE had developed in about half of patients with DPD of NSCLC and only a small portion of patients needed invasive intercostal drainage for the management symptomatic MPE. MPE in DPD did not affect the survival in NSCLC patients.

## Author contributions

**Conceptualization:** In Kyu Park, Woojung Kim.

**Data curation:** Woojung Kim, Samina Park.

**Formal analysis:** Woojung Kim.

**Investigation:** Woojung Kim.

**Methodology:** In Kyu Park, Samina Park.

**Project administration:** Woojung Kim.

**Resources:** In Kyu Park, Chang Hyun Kang, Young Tae Kim.

**Software:** Woojung Kim.

**Supervision:** Samina Park, Chang Hyun Kang, Young Tae Kim.

**Validation:** Samina Park, Young Tae Kim.

**Visualization:** Woojung Kim.

**Writing – original draft:** Woojung Kim.

**Writing – review & editing:** In Kyu Park, Chang Hyun Kang, Young Tae Kim.

Woojung Kim orcid: 0000-0001-9530-8638.
